# Adoption, usage patterns, and perceptions of DeepSeek among medical interns: Evidence from a Chinese Teaching Hospital

**DOI:** 10.1371/journal.pone.0362171

**Published:** 2026-11-02

**Authors:** Tao Zhang, Dong Pan, Zihao Zhang, Xueping Zhang, Ming Wang

**Affiliations:** 1 Department of Urology, The Second Affiliated Hospital of Anhui Medical University, Hefei, China; 2 The First Affiliated Hospital, and College of Clinical Medicine of Henan University of Science and Technology, Luoyang, China; 3 Department of Urology, Weifang People’s Hospital, Weifang, China; University of Porto Faculty of Medicine: Universidade do Porto Faculdade de Medicina, PORTUGAL

## Abstract

Artificial intelligence (AI)–driven chatbots based on large language models are increasingly entering medical education, but evidence on the real-world use of domestic systems such as DeepSeek among Chinese medical interns is still scarce. This cross-sectional study investigated the prevalence, patterns and perceptions of DeepSeek use among medical interns at the Second Affiliated Hospital of Anhui Medical University. A total of 634 interns from clinical medicine, preventive medicine and nursing completed an anonymous questionnaire. Overall, about one-third of interns reported using DeepSeek. Among users, most engaged with the tool regularly, with over half using it several times per week. DeepSeek was primarily employed for completing assignments, searching for information, translation and polishing of written texts, while a smaller subset of users reported using it for data analysis. Attitudes toward DeepSeek were mainly neutral or optimistic, and across majors most users perceived its advantages and disadvantages as broadly balanced, while only a minority considered the disadvantages to outweigh the advantages. After adjustment, residence and major remained associated with DeepSeek use, whereas age and sex were not. Urban interns were more likely to report using DeepSeek than rural interns. Compared with clinical medicine interns, preventive medicine interns were less likely to report use, whereas nursing interns were more likely to do so. Among users, overall attitudes differed across majors, while usage frequency and the perceived balance of advantages and disadvantages did not. These findings provide descriptive evidence on the use and evaluation of DeepSeek among medical interns and may inform future institutional guidance on responsible use, verification of AI-generated information and academic integrity.

## 1. Introduction

Artificial intelligence (AI)–driven chatbots are advancing rapidly and are increasingly explored as tools for medical education [1]. They are regarded as flexible digital assistants that can support multiple core teaching and learning tasks [2]. For example, AI chatbots can assist in the design and continuous updating of instructional materials for undergraduate, postgraduate, and continuing medical education, and can automatically generate assessment items targeting both foundational knowledge and applied clinical competencies [3]. They can also simulate realistic conversational scenarios, functioning as virtual patients for practicing doctor–patient communication, and can be embedded into interactive platforms to enhance multimedia learning experiences [4]. Owing to these capabilities, AI chatbots are gaining recognition as useful aids for examination preparation and for the rapid retrieval of essential information. Central to many of these systems are large language models (LLMs), which represent a transformative technology in this domain. LLMs possess advanced abilities in language understanding and context-aware response generation, and their use in medical education is expanding [5]. Trained on extensive and diverse corpora, these models can interpret complex clinical queries and provide structured, up-to-date information [6]. Such functions are particularly advantageous in educational settings, where timely and well-organized content supports learners and healthcare professionals in refining clinical reasoning and making informed decisions.

Medical education is characterized by a continually expanding knowledge base and a demanding curriculum that requires students to synthesize information from basic sciences, clinical disciplines, and rapidly evolving guidelines [7]. Traditionally, this learning process has been anchored in printed textbooks, structured lectures, and supervised clinical rotations. In parallel with the widespread diffusion of smartphones and high-speed internet, however, medical students have increasingly turned to digital resources to cope with information overload and time pressure [8]. Among these resources, LLM-based chatbots have begun to occupy a distinctive niche, functioning as on-demand helpers for clarifying difficult concepts, summarizing lengthy materials, drafting or polishing written assignments, and rehearsing examination-style questions. For many students, such tools offer a perception of individualized support that is not always feasible within conventional teaching formats, especially when faculty time is limited and class sizes are large [9]. The growing presence of these systems in students’ day-to-day learning has nonetheless prompted debate within the medical education community. On the one hand, AI-assisted querying may promote efficient access to definitions, mechanisms, and differential diagnoses, and can encourage learners to revisit topics that they might otherwise avoid due to their complexity. On the other hand, current large language models generate text based on statistical patterns rather than verified expert consensus, and their outputs can contain inaccuracies, omissions, or contextually inappropriate statements when applied to specific clinical problems [10]. There is concern that medical students, especially at earlier stages of training, may accept such responses uncritically, with possible implications for the development of critical appraisal skills, independent reasoning, and academic integrity [11]. These considerations underscore the importance of examining not only whether medical students use AI chatbots, but also the frequency, purposes, and perceived consequences of such use in real educational settings.

In many contexts, the use of internationally known LLM-based systems such as ChatGPT is not as straightforward as often assumed. Access may be limited by regulatory policies, network constraints, or the cost of subscription services, and these factors together reduce the feasibility of relying on such tools in routine learning [12]. In mainland China, this situation has encouraged the development and adoption of domestic large language models, among which DeepSeek has become a commonly used platform on university campuses [13]. For most students, DeepSeek is relatively easy to access, works well with Chinese-language input, and is embedded in local digital ecosystems that they already use for study and communication. These features lower the practical barriers to use and make it a natural option for day-to-day learning tasks, including looking up medical information, generating brief summaries, drafting or revising short texts, and preparing for examinations within a familiar linguistic and cultural environment [14]. Although DeepSeek is now widely visible in student life, systematic data on its role in medical education are still very limited. Most of the available literature on AI-assisted learning has concentrated on general perceptions of artificial intelligence or on the use of international systems, while domestic LLMs in China have received far less attention.

The present study aimed to provide a focused examination of DeepSeek use among medical interns in China. We conducted a cross-sectional survey among medical interns from three training pathways—clinical medicine, preventive medicine, and nursing—to assess DeepSeek use, usage patterns, and overall evaluations in these medical interns. By characterizing real-world usage patterns, this study seeks to generate evidence that may support the responsible and context-appropriate integration of large language models into medical training.

## 2. Methods

### 2.1. Design and participants

This cross-sectional study was conducted from June to October 2025 at the Second Affiliated Hospital of Anhui Medical University, with undergraduate medical interns at the hospital serving as the study participants. The inclusion criteria were as follows: (1) full-time undergraduate students completing clinical internships at the hospital during the survey period; (2) students majoring in clinical medicine, preventive medicine, or nursing; (3) able to understand and independently complete the questionnaire; (4) able to fully complete demographic information and core questions regarding DeepSeek usage. Undergraduate interns not majoring in one of the three disciplines mentioned above, those who refused to participate or withdrew midway, or those whose submitted questionnaires contained incomplete demographic information or omitted answers to the core questions regarding DeepSeek usage were excluded. Given that this study was exploratory in nature, no formal prior sample size calculation was performed.

A total of 657 questionnaires were returned. Of these, 23 respondents provided only partial information or completed only the demographic section, therefore these questionnaires were excluded from the analysis. The final analytic sample therefore comprised 634 medical interns. Ethical approval was waived by the Ethics Committee of the Second Affiliated Hospital of Anhui Medical University because the study posed minimal risk and employed an anonymized data-collection procedure in which no personally identifiable information was obtained or retained. Informed consent was obtained from all participants before they completed the questionnaire.

### 2.2 Data collection and questionnaire

Data collection was carried out during scheduled teaching in the hospital. A self-administered, anonymous questionnaire was used to assess the application of the DeepSeek large language model among undergraduate medical interns. Before distributing the questionnaire, the investigators briefly explained the purpose and significance of the study and emphasized that participation was voluntary and that responses would be kept confidential and used only for research.

The questionnaire was designed with reference to previous studies on artificial intelligence and large language models in medical education and was adapted to the practical context of DeepSeek use among medical interns [15]. The questionnaire contained nine items. Items 1–4 collected demographic information, including age, sex, place of residence, and major. Item 5 assessed whether participants had ever used DeepSeek for learning. Items 6–9 were completed only by participants who answered “yes” to item 5 and assessed frequency of use, primary learning purpose, overall attitude and perceived balance of advantages and disadvantages, respectively. Except for age, all items were categorical variables; the application-purpose question required participants to select only one primary purpose.

With reference to previous studies [15,16], overall attitude was measured using three direct response options with explanatory definitions: optimistic, defined as intending to maintain or increase the current frequency of DeepSeek use in the future; neutral, defined as using DeepSeek depending on future academic needs; and pessimistic, defined as not intending to continue using DeepSeek in the future. The perceived balance between advantages and disadvantages was also measured using three direct response options with explanatory definitions, and the question stem explicitly defines the scope of advantages and disadvantages that participants should consider when making their judgment. These variables were based on participants’ direct self-reported responses. The full questionnaire, including the wording of the questions, response options, and explanatory definitions, is provided in S1 File. All questionnaires were completed without collecting any personally identifiable information.

### 2.3. Statistical analysis

Continuous variables were summarized using medians and interquartile ranges (IQRs) when distributions were non-normal, and categorical variables were presented as frequencies and percentages. Differences in categorical variables were examined using Chi-square tests. Group comparisons were conducted using Mann-Whitney U test. A two-sided p value of < 0.05 was considered to indicate statistical significance. A multivariable binary logistic regression analysis was also performed in this study, adjusted odds ratios (ORs) and 95% confidence intervals (CIs) were reported.

## 3. Results

### 3.1. Participant characteristics

A total of 634 medical interns from the Second Affiliated Hospital of Anhui Medical University were included in the final analysis. The median age was 22.0 years (interquartile range, 21.0–22.0). Overall, 315 students (49.7%) reported a rural residence and 319 (50.3%) an urban residence. The sex distribution was approximately balanced, with 310 males (48.9%) and 324 females (51.1%). With respect to academic major, 346 interns (54.6%) were enrolled in clinical medicine, 194 (30.6%) in preventive medicine, and 94 (14.8%) in nursing (Table 1).

**Table 1:** Participant demographics.

Variables	Total (n = 634)	Used DeepSeek		P value
		Yes (n = 228)	No (n = 406)	
Age, Median (IQR)	22.0, (21.0–22.0)	22.0, (21.0–22.0)	22.0, (21.0–23.0)	P = 0.069
Residence, No. (%)				
Rural	315 (49.7%)	91 (39.9%)	224 (55.2%)	P < 0.001
Urban	319 (50.3%)	137 (60.1%)	182 (44.8%)	
Sex, No. (%)				
Male	310 (48.9%)	110 (48.2%)	200 (49.3%)	P = 0.806
Female	324 (51.1%)	118 (51.8%)	206 (50.7%)	
Major, No. (%)				
Clinical medicine	346 (54.6%)	125 (54.8%)	221 (54.4%)	P < 0.001
Preventive medicine	194 (30.6%)	55 (24.1%)	139 (34.2%)	
Nursing	94 (14.8%)	48 (21.1%)	46 (11.3%)	

Note: Data are presented as median (IQR) or counts (%). IQR, interquartile range.

Among all participants, 228 interns (36.0%) reported having used DeepSeek, whereas 406 (64.0%) had not. The median age did not differ between users and non-users. Compared with students from rural areas, those with an urban residence were more likely to report using DeepSeek for learning. The proportion of users also varied significantly by major: 125 of 346 clinical medicine interns (36.1%) and 55 of 194 preventive medicine interns (28.4%) reported using DeepSeek, whereas 48 of 94 nursing interns (51.1%) did so, indicating a particularly high uptake in the nursing group. In contrast, the proportion of DeepSeek users did not differ significantly between male and female students (Table 1).

### 3.2. Frequency of DeepSeek use

Among the 228 interns who reported using DeepSeek, more than half (123, 53.9%) indicated that they used the tool several times per week, and 28 (12.3%) reported daily use. A further 53 students (23.2%) used DeepSeek approximately once per week, while only 24 (10.5%) described their use as occasional (Table 2). Thus, the majority of users engaged with DeepSeek at least weekly.

**Table 2:** Usage frequency, attitudes, and perceived pros and cons balance of students toward DeepSeek.

Variables	Students (n = 228)
Frequency, No. (%)	
Every day	28 (12.3%)
Several times a week	123 (53.9%)
Once a week	53 (23.2%)
Occasionally	24 (10.5%)
Attitude, No. (%)	
Optimistic	95 (41.7%)
Neutral	102 (44.7%)
Pessimistic	31 (13.6%)
Pros and cons of using DeepSeek, No. (%)	
Pros outweigh cons	64 (28.1%)
Pros and cons were balanced	147 (64.5%)
Cons outweigh pros	17 (7.5%)

Note: Data are presented as counts (%).

When examined by major, generally similar patterns were observed. Among clinical medicine interns who used DeepSeek, 18 (14.4%) students reported using it every day, 65 (52.0%) several times per week, 30 (24.0%) about once per week, and 12 (9.6%) only occasionally. In preventive medicine, 6 (10.9%) students reported daily use, 32 (58.2%) used it several times per week, 12 (21.8%) once per week, and 5 (9.1%) occasionally. In nursing, 4 (8.3%) interns reported daily use, 26 (54.2%) used DeepSeek several times per week, 11 (22.9%) once per week, and 7 (14.6%) occasionally. Across all three majors, the largest group consisted of students who used DeepSeek several times per week, and only a small proportion in each major reported purely occasional use.

### 3.3. Attitudes toward DeepSeek

With regard to overall attitudes toward DeepSeek, 95 of the 228 users (41.7%) expressed an optimistic view, 102 (44.7%) were neutral, and 31 (13.6%) reported a pessimistic attitude (Table 2).

Among clinical medicine interns, 59 (47.2%) of 125 users were optimistic about DeepSeek, 48 (38.4%) were neutral, and 18 (14.4%) were pessimistic. In preventive medicine, 27 (49.1%) of 55 users reported an optimistic attitude, 22 (40.0%) were neutral, and 6 (10.9%) were pessimistic. Nursing interns tended to be more reserved, among 48 users, 9 (18.8%) expressed optimism, 32 (66.7%) were neutral, and 7 (14.6%) were pessimistic.

### 3.4. Perceived balance of advantages and disadvantages

When asked to weigh the advantages and disadvantages of using DeepSeek in their learning, most users (147, 64.5%) considered the pros and cons to be roughly balanced. Sixty-four students (28.1%) believed that the benefits outweighed the drawbacks, whereas 17 (7.5%) felt that the disadvantages were predominant (Table 2).

Among clinical medicine interns who used DeepSeek, 37 (29.6%) students judged that the benefits outweighed the drawbacks, 83 (66.4%) perceived the pros and cons as roughly equal, and 5 (4.0%) considered the disadvantages to predominate. In preventive medicine, 16 (29.1%) users reported that the pros outweighed the cons, 31 (56.4%) perceived a balance, and 8 (14.5%) believed that the cons outweighed the pros. In nursing, 11 (22.9%) of 48 users felt that the benefits were greater than the drawbacks, 33 (68.8%) perceived them as balanced, and 4 (8.3%) believed that the drawbacks predominated.

### 3.5. Application scenarios of DeepSeek

Students who used DeepSeek reported employing it for a range of learning-related tasks (Table 3). The most commonly reported application was completing assignments, which was mentioned by 63 users (27.6%). Information search was also frequent (51 students, 22.4%), followed by translation tasks (41, 18.0%) and paper polishing (39, 17.1%). A smaller but noteworthy proportion of users (34, 14.9%) reported using DeepSeek for data analysis. These patterns suggest that, in this cohort of medical interns, DeepSeek functioned not only as a general information retrieval tool but also as an auxiliary aid for coursework and written work, with more specialized uses such as data analysis adopted by a subset of students.

**Table 3:** Primary learning purpose for using DeepSeek.

Variables	Students (n = 228)
Applications, No. (%)	
Information search	51 (22.4%)
Translation	41 (18.0%)
Paper polishing	39 (17.1%)
Completing assignments	63 (27.6%)
Data analysis	34 (14.9%)

Note: Data are presented as counts (%).

### 3.6. Factors associated with DeepSeek use

A multivariable binary logistic regression analysis was performed to examine whether the associations between demographic characteristics and DeepSeek use remained after mutual adjustment (Table 4). Compared with interns from rural areas, those from urban areas had higher odds of using DeepSeek (adjusted OR=2.019, 95% CI: 1.436–2.838, P < 0.001). Major was also associated with DeepSeek use. Compared with clinical medicine interns, preventive medicine interns had lower odds of using DeepSeek (adjusted OR=0.608, 95% CI: 0.408–0.907, P = 0.015), whereas nursing interns had higher odds of use (adjusted OR=1.732, 95% CI: 1.002–2.995, P = 0.049). Neither age nor sex was significantly associated with DeepSeek use after adjustment.

**Table 4:** Association between demographic characteristics and DeepSeek use among medical interns.

Variable	DeepSeek use			
	Adjusted B	SE	Adjusted OR (95% CI)	P value
Age (per 1-year increase)	−0.078	0.094	0.925 (0.769–1.112)	0.406
**Sex**				
Female	Reference			
Male	0.153	0.183	1.165 (0.814–1.667)	0.403
**Residence**				
Rural	Reference			
Urban	0.703	0.174	2.019 (1.436–2.838)	<0.001
**Major**				
Clinical medicine	Reference			
Preventive medicine	−0.497	0.204	0.608 (0.408–0.907)	0.015
Nursing	0.549	0.279	1.732 (1.002–2.995)	0.049

Note: B, regression coefficient; SE, standard error; OR, odds ratio; CI, confidence interval. The dependent variable was DeepSeek use (yes = 1, no = 0); female sex, rural residence, and clinical medicine were the reference categories.

### 3.7. Comparison of usage patterns and perceptions across majors

Among the 228 DeepSeek users, usage frequency did not differ significantly across the three majors (Table 5). However, the distribution of attitudes differed significantly by major. Nursing interns reported a lower proportion of optimistic attitudes and a higher proportion of neutral attitudes than clinical medicine and preventive medicine interns. No significant between-major difference was observed in the perceived balance of advantages and disadvantages.

**Table 5:** Comparison of DeepSeek usage patterns and perceptions across majors among users.

Variable	Clinical medicine (n = 125)	Preventive medicine (n = 55)	Nursing (n = 48)	χ²	P value
**Frequency, No. (%)**				2.463	0.873
Every day	18 (14.4)	6 (10.9)	4 (8.3)		
Several times a week	65 (52.0)	32 (58.2)	26 (54.2)		
Once a week	30 (24.0)	12 (21.8)	11 (22.9)		
Occasionally	12 (9.6)	5 (9.1)	7 (14.6)		
**Attitude, No. (%)**				14.640	**0.006**
Optimistic	59 (47.2)	27 (49.1)	9 (18.8)		
Neutral	48 (38.4)	22 (40.0)	32 (66.7)		
Pessimistic	18 (14.4)	6 (10.9)	7 (14.6)		
**Pros and cons, No. (%)**				7.107	0.130
Advantages outweigh disadvantages	37 (29.6)	16 (29.1)	11 (22.9)		
Approximately balanced	83 (66.4)	31 (56.4)	33 (68.8)		
Disadvantages outweigh advantages	5 (4.0)	8 (14.5)	4 (8.3)		

Note: Values are n (%). Pearson χ^2^ tests were used.

## 4. Discussion

This study provides empirical descriptions of DeepSeek use among medical interns in China, elucidating the perceptions and utilization of large language models among medical interns. Several notable findings emerged. First, only about one-third of the interns reported using DeepSeek, indicating that its use was not universal in this cohort. Second, among those who did use DeepSeek, usage tended to be frequent, with the majority engaging with the tool at least several times per week. Third, most users reported neutral or optimistic attitudes toward DeepSeek, and only a small proportion reported pessimistic attitudes. After adjustment, residence and major remained associated with DeepSeek use, whereas age and sex were not significantly associated with its use. Among users, the distribution of overall attitudes differed significantly across majors, while no significant between-major differences were found in usage frequency or the perceived balance of advantages and disadvantages.

The overall adoption rate in our cohort appeared lower than that reported in studies conducted in Western educational contexts, where tools such as ChatGPT have been rapidly incorporated into students’ learning routines [17]. However, such comparisons should be interpreted cautiously because the platforms, study populations, and educational settings differ. Potential contextual explanations include access restrictions and the limited availability of international LLM platforms in China, which may encourage reliance on domestic models such as DeepSeek [18]. Nevertheless, DeepSeek is comparatively new, and its integration into medical curricula or institutional training practices has not yet been formalized, which may partly explain why many interns have not used it despite being aware of its existence. Additionally, the clinical internship period is highly structured and time-intensive, leaving limited opportunity for students to actively experiment with new digital tools unless they perceive clear learning benefits. However, our survey did not assess platform accessibility, curricular integration, internship workload, or reasons for non-use. These factors should therefore be regarded only as possible explanations requiring further investigation.

Among students who used DeepSeek, the relatively high frequency of engagement, particularly weekly or multiple times per week, indicated regular engagement with the tool in this subgroup. This pattern is broadly consistent with findings from studies of ChatGPT and similar systems, in which students often use LLMs to clarify difficult concepts, reduce cognitive load, and assist with summarization or translation tasks [19]. In our study, the most common applications included completing assignments, conducting information searches, and supporting English translation or text refinement, and these represent common academic activities in medical training.

Attitudes toward DeepSeek were generally neutral or optimistic, although their distribution differed significantly across majors. Although educators have raised concerns that LLMs may encourage superficial understanding or promote overreliance on automated content generation [20], only 13.6% of users in our study reported a pessimistic attitude. The finding that nursing interns exhibited a lower proportion of optimistic responses and a higher proportion of neutral responses than clinical medicine and preventive medicine interns indicates a comparatively reserved attitude toward future use. Differences in educational and practice contexts across disciplines may be relevant to this variation [21], though the specific reasons require further investigation. These findings highlight the importance of considering discipline-specific perspectives when interpreting students’ acceptance of AI-assisted learning tools.

Perceptions of DeepSeek’s advantages and disadvantages further illustrate how students conceptualize the role of LLMs in medical education. Most users across all majors regarded the benefits and drawbacks as balanced rather than strongly positive or negative [22]. Moreover, no significant difference was observed across majors, indicating broadly similar evaluations of the balance between advantages and disadvantages among the three groups.

Pan and Ni reported that about one-third of 1718 medical undergraduates had heard of and used large language models [15], a proportion similar to that of students in this study who used DeepSeek for learning. Similarly, Tao et al. found that 28.72% of 693 medical students from 57 universities in China had used AI chatbots to assist in learning [23]. In contrast, Hu et al. reported that 62.9% of 1133 medical students used ChatGPT in their medical studies [16]. Zhang et al. found that only 5.1% of medical students had never been exposed to AI language generation models, although more than half of the students used them less than once a week [24]. The differences in usage reported in the studies may reflect differences in assessment platforms, definitions of use, questionnaire formats, participating groups, and educational environments. In terms of learning uses, Hu et al. pointed out that information retrieval and completing assignments are the most common applications of ChatGPT [16]. Tao et al. reported that students often use it to obtain medical information and improve learning efficiency [23]. Zhang et al.‘s research also found that these models are mainly used to solve problems related to medical courses and academics [24]. These findings are largely consistent with our observations; however, since our questionnaire was in single-choice format, the results need cautious comparison and interpretation.

Taken together, these findings highlight preliminary practical implications for medical education in China. As LLMs such as DeepSeek become more capable and widely accessible, their use in clinical learning environments is likely to expand. The reported use of DeepSeek for assignments and paper polishing identifies acceptable assistance, disclosure, and academic integrity as areas that institutions may wish to clarify in future guidance. Similarly, its use for information retrieval and data analysis suggests that source verification, critical appraisal of generated content, and appropriate methodological oversight may warrant attention. AI literacy and the critical appraisal of AI-generated content may therefore be considered in future curricular planning [25]. For institutions, understanding how students currently use LLMs can inform decisions about policy development, academic integrity guidelines, and incorporation of AI technologies into teaching strategies [26]. The observed differences in attitudes across majors also indicate that disciplinary context should be considered when exploring students’ needs and developing future guidance, rather than assuming that a uniform approach will be appropriate for all medical disciplines [27].

However, some limitations should be acknowledged. First, the study was conducted at a single teaching hospital, which may limit generalizability to other institutions with different educational structures or student populations. Second, the cross-sectional design captures usage and attitudes at one point in time; longitudinal studies are needed to understand how perceptions evolve as DeepSeek and other LLMs continue to advance. Third, the questionnaire was adapted from previously published survey studies and was not formally piloted; therefore, formal validity and reliability testing could not be performed. Finally, consistent with previous studies, attitudes and perceived advantages and disadvantages were assessed using brief global categorical items rather than validated multi-item psychometric scales. Therefore, these findings should be interpreted as descriptive overall perceptions rather than validated measures of specific attitudes or particular perceived benefits and concerns.

## 5. Conclusion

Among medical interns, DeepSeek is used by a minority but, when adopted, is integrated into learning on a regular basis. Users generally reported neutral to optimistic views and perceived the advantages and disadvantages of DeepSeek as broadly balanced. These findings indicate that DeepSeek currently functions as an auxiliary learning aid and underscore the need for structured guidance on its appropriate use in medical education.

## Supporting information

S1 File Appendix 1: Questionnaire on DeepSeek use among medical interns. (DOCX)
