# Supplementary material for: Adoption, usage patterns, and perceptions of DeepSeek among medical interns: Evidence from a Chinese Teaching Hospital
Source: PLoS One. 2026 Nov 2;21(11):e0362171. doi: 10.1371/journal.pone.0362171 (PMC13726908; doi:10.1371/journal.pone.0362171)
Supplement: Supplementary file 1 [file pone.0362171.s001.docx]

**Appendix 1** Questionnaire on DeepSeek Use Among Medical Interns

| **Questions** | **Response Options** |
| --- | --- |
| 1. Age | ___ years |
| 2. Sex (choose one) | - Male - Female |
| 3. Place of residence (choose one) | - Rural - Urban |
| 4. Major (choose one) | - Clinical medicine - Preventive medicine - Nursing |
| 5. Have you ever used DeepSeek for learning or study during your medical internship? (choose one) | - Yes - No |
| 6. How often do you use DeepSeek for learning or study? (choose one)  Only participants who answer "Yes" to Question 5 answer this question. | - Every day - Several times per week - Once per week - Occasionally |
| 7. What is your primary purpose for using DeepSeek in learning? (choose one)  Only participants who answer "Yes" to Question 5 answer this question. | - Information search - Translation - Paper polishing - Completing assignments - Data analysis |
| 8. What is your overall attitude toward using DeepSeek in medical learning? (choose one)  Only participants who answer "Yes" to Question 5 answer this question. | - Optimistic: I intend to maintain or increase my current frequency of using DeepSeek in the future. - Neutral: I will use DeepSeek depending on my future academic needs. - Pessimistic: I do not intend to continue using DeepSeek in the future. |
| 9. Overall, how do you judge the balance between the advantages and disadvantages of using DeepSeek for learning? (choose one)  Only participants who answer "Yes" to Question 5 answer this question. When answering this question, please consider both possible learning benefits, such as improved efficiency, information retrieval, explanation of difficult concepts, translation, writing assistance, and research-related support, and possible concerns, such as inaccurate or fabricated information, lack of verifiable sources, privacy risks, bias, overreliance, academic integrity, and effects on critical thinking. | - Advantages outweigh disadvantages: I believe that, overall, the learning benefits of using DeepSeek are greater than its potential disadvantages or risks. - Advantages and disadvantages are balanced: I believe that DeepSeek has both useful learning value and potential disadvantages, and neither clearly outweighs the other. - Disadvantages outweigh advantages: I believe that, overall, the potential disadvantages or risks of using DeepSeek are greater than its learning benefits. |
